# Minimal change disease in treatment-naïve hepatitis C virus infection: A case report and literature review 

**DOI:** 10.5414/CNCS111506

**Published:** 2025-01-10

**Authors:** Juliano Alhaddad, Hazim Allos, Dimo Dimitrov, Claudia M. Nader, Helmut G. Rennke, Bertrand L. Jaber

**Affiliations:** 1 Department of Medicine,; 2 Division of Infectious Diseases,; 3 Division of Nephrology, St. Elizabeth’s Medical Center, Boston, MA,; 4 Department of Medicine, Tufts University School of Medicine,; 5 Department of Pathology, Brigham and Women’s Hospital, and; 6 Department of Pathology, Harvard Medical School, Boston, MA, USA

**Keywords:** minimal change disease, nephrotic syndrome, hepatitis C virus infection

## Abstract

Minimal change disease (MCD) accounts for 10 – 15% of idiopathic nephrotic syndromes in adults. Chronic hepatitis C virus (HCV) infection is rarely ascribed as a cause of MCD and was previously associated with interferon-based therapy. MCD in treatment-naïve chronic HCV infection is extremely rare, with only 3 cases reported in the literature. We report on a 67-year-old woman presenting with acute nephrotic syndrome and severe acute kidney injury requiring short-term dialysis. She was initially treated empirically with glucocorticoids and underwent a kidney biopsy that revealed MCD with evidence of acute tubular necrosis and mild focal acute interstitial nephritis. An extensive work-up was only significant for the presence of anti-HCV antibody with an elevated HCV viral load of genotype 1b. Her kidney function recovered, and she was discharged on an oral prednisone course with a planned taper. 4.5 months later, her HCV infection was treated with ledipasvir and sofosbuvir, and she achieved sustained virological response. The nephrotic syndrome remained in remission 24 months after initial presentation. This is a unique case where sustained remission of both the nephrotic syndrome and the HCV infection were achieved with glucocorticoids and direct antiviral agents, respectively.

## Introduction 

Minimal change disease (MCD) is the most common cause of the nephrotic syndrome in children (70 – 90% of cases), but it accounts for 10 – 15% of idiopathic cases in the adult population [1]. The clinical syndrome typically entails heavy proteinuria with an acute sudden onset of edema [2]. Acute kidney injury can develop, but it is more frequent in adults [1, 2, 3]. Secondary causes of MCD include neoplastic diseases, infections, and drug exposures [4]. Hepatitis C virus (HCV) infection is usually associated with cryoglobulinemic membranoproliferative glomerulonephritis, and less frequently, with other glomerular diseases such as membranous nephropathy [5]. However, MCD is rarely seen among patients with HCV infection receiving interferon therapy, which used to be first-line therapy [6, 7, 8]. Indeed, a known but rare adverse effect of interferon therapy includes the development of MCD as well as focal segmental glomerulosclerosis [6, 8, 9]. 

We describe the case of an adult woman presenting with severe acute kidney injury requiring temporary dialysis and found to have nephrotic range proteinuria and a new diagnosis of genotype 1b HCV infection, with biopsy-proven MCD. Remission of the nephrotic syndrome was initially achieved with prednisone and was maintained following successful treatment of the HCV infection with direct-acting antiviral agents (DAAs). We conducted a literature review of previous cases of MCD in patients with treatment-naïve HCV infection and summarized the treatment and disease course. 

## Case presentation 

The patient is a 67-year-old White woman who presented to the hospital with a 2-week history of bilateral knee pain following a fall at home. Her past medical history was significant for hypertension, atrial fibrillation, hypothyroidism, and chronic obstructive pulmonary disease (on home oxygen). Her review of systems was notable for dizziness, generalized body ache, chronic cough, and dyspnea. She denied use of non-steroidal anti-inflammatory drugs. Physical examination revealed bibasilar rales on lung auscultation and anasarca with bilateral lower extremity edema and ascites. The chest X-ray revealed bilateral lung infiltrates and pleural effusions. She had stage 3 acute kidney injury with an initial serum creatinine of 7.0 mg/dL, up from a normal baseline value, which peaked at 8.1 mg/dL. Serum albumin level was low at 2.5 g/dL. The urinalysis revealed a specific gravity of 1.023, pH of 5, greater than 1,000 mg/dL of protein, and moderate blood. The urine sediment demonstrated non-dysmorphic red blood cells, hyaline casts, fine granular casts, and degenerated cellular casts. The random urine total protein was measured twice and was greater than 600 mg/dL, as a result, the urine total protein-to-creatinine ratio could not be calculated. A kidney ultrasound revealed no obstructive uropathy, and the kidneys were normal in size and had normal echogenicity. 

Her clinical presentation was concerning for rapidly progressive glomerulonephritis, and she was empirically started on pulse methylprednisolone (1 g intravenously daily for 3 days followed by 500 mg intravenously on the 4^th^ day). Two days after presentation, she was initiated on intermittent hemodialysis for refractory volume overload and worsening metabolic acidosis. An extensive serological and immunological work up was unrevealing, including non-reactive titers for antinuclear antibody, anti-double stranded DNA antibody, anti-neutrophil cytoplasmic antibodies, anti-myeloperoxidase and anti-proteinase 3 antibodies, anti-phospholipase A2 receptor antibody, and anti-glomerular basement membrane antibody. Complement levels were normal, rheumatoid factor screen was negative, and cryoglobulins were undetected. Serum protein electrophoresis revealed no monoclonal protein but a low serum albumin level. Her hepatitis B virus, human immunodeficiency virus, and syphilis screen were all negative. However, the HCV antibody titer was reactive, and the HCV RNA viral load was elevated at 1,7800,000 IU/mL. 

**Figure 1.**
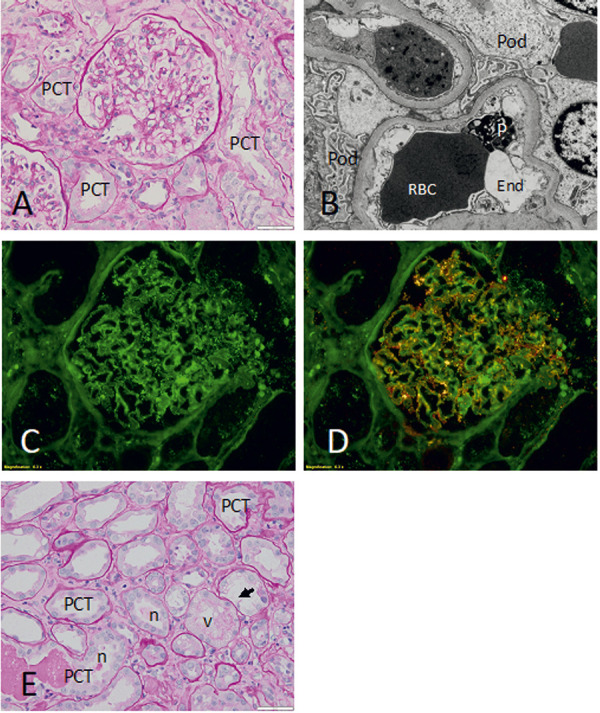
A: The glomerulus reveals a normal histology with delicate capillary walls and normal cellularity. (PAS; Magnification × 230); B: The electron microscopy shows diffuse effacement of the foot processes of the glomerular epithelial cells or podocytes with microvillous changes of the cell surface (Original magnification on the EM image, × 6,000); C: The immunofluorescence microscopy shows “fine dusting” of IgG deposited over the podocytes and along the glomerular capillary walls (FITC-labelled anti-IgG; Magnification × 345); D: The double stain with rhodamine-labelled anti-nephrin antibodies (red) and FITC-labelled anti-IgG antibodies (green) shows in yellow the sites where the two signals overlap, indicating complexes of nephrin with human IgG (double immunofluorescence microscopy stain as described above; Magnification × 345); E: Cortical tubules reveal diffuse flattening of the proximal convoluted tubule (PCT) epithelial cells, focal necrosis (n), vacuolization of better-preserved tubules (v), and focal loss of the epithelium (arrow) (PAS; Magnification × 230). PCT = proximal convoluted tubule; Pod = podocyte or glomerular visceral epithelial cell; P = platelets; RBC = red blood cell; End = endothelium, focally swollen; n = necrosis, cell debris; v = vacuolization of epithelial cells.

Given the unexplained kidney failure, she underwent a percutaneous kidney biopsy. The kidney biopsy included 23 glomeruli, 1 with global sclerosis. The viable glomeruli revealed a normal morphology by light microscopy (Figure 1A), the electron microscopy showed extensive podocyte injury with diffuse effacement of the foot process interdigitations, extensive loss of filtration slit diaphragms, and focal detachment of foot processes from the basal membrane (Figure 1B). The immunofluorescence microscopy revealed “fine-dusting” of IgG deposits on the cell surface of podocytes (Figure 1C). These features are diagnostic of a diffuse podocytopathy, namely MCD. Furthermore, the co-localization of IgG and nephrin in the dual stain was strongly suggestive of an anti-nephrin antibody-mediated process (Figure 1D). There was also evidence of acute tubular injury with focal tubular necrosis and mild focal acute interstitial nephritis (Figure 1E). Out of the 23 sampled glomeruli, only 1 glomerulus (4%) had global sclerosis, a level of sclerosis within the normal limit of age-associated glomerular obsolescence, effectively ruling out any focal segmental glomerulosclerosis. 

Following pulse methylprednisolone therapy, her kidney function recovered, and hemodialysis was discontinued after 7 daily sessions. She was discharged from the hospital on an oral course of prednisone 60 mg once daily, with an initial plan for a 16-week taper. At the time of hospital discharge, her serum creatinine was 1.6 mg/dL. Two months later, her serum creatinine was 0.83 mg/dL, and a random urine total protein to creatinine ratio was 510 mg/g. 

She was formally evaluated for her genotype 1b chronic HCV infection. She had no evidence of advanced liver fibrosis and was started on DAA for her chronic HCV infection with ledipasvir/sofosbuvir, 4.5 months after her initial presentation. At that time, she was on a tapered prednisone dose of 10 mg daily. She successfully completed a 12-week antiviral treatment course. The HCV RNA remained undetectable 1.5 years later, confirming a sustained virological response. Unfortunately, the patient was inconsistent with her clinic visits, resulting in a longer than planned prednisone taper. Six months after her initial presentation, while on prednisone 5 mg every other day, the urine total protein-to-creatinine ratio remained normal at 134 mg/g, consistent with full remission of the nephrotic syndrome. On her most recent visit with her primary care provider, almost 2 years from the time of her initial presentation, her urine albumin-to-creatinine ratio was less than 30 mg/g. 

## Discussion 

The association of chronic HCV infection and kidney disease is well described in the literature. Hepatitis C infection has primarily been associated with glomerulonephritis, namely mixed cryoglobulinemic vasculitis with a membranoproliferative pattern of injury on renal histopathology [5]. The clinical presentation is usually that of a nephritic syndrome, however, a combined nephritic-nephrotic syndrome has been recognized [6]. Other HCV-associated glomerulopathies include membranous nephropathy, focal segmental glomerulosclerosis, immunoglobulin A (IgA) nephropathy, and fibrillary glomerulonephritis [5, 10]. 

MCD, however, is very rarely associated with HCV infection, with only a few cases reported to date. Minimal change disease was initially reported in patients with HCV infection receiving interferon-based therapy, where it was postulated to be secondary to interferon-mediated immune system activation, as it has been also described in other conditions treated with interferon [7, 8, 11]. In a case series from Japan, over the period of 1992 – 2008, among 68 patients with a reactive anti-HCV antibody titer and detectable HCV RNA who underwent a kidney biopsy for persistent proteinuria, hematuria, and/or renal dysfunction, only 1 patient who was 46 years old with normal kidney function (serum creatinine 0.7 mg/L) and minimal proteinuria (0.2 gm/day) was diagnosed with MCD [12]. 

The association between MCD and HCV in treatment-naïve patients is even rarer. A review of the literature identifies three additional case reports that are summarized in Table 1. In 2007, Aoyama et al. [13] reported the case of a 49-year-old Japanese man presenting with acute-onset anasarca and found to have nephrotic range proteinuria (13.6 g/day), acute kidney injury, hemolytic anemia, and thrombocytopenia. The kidney biopsy did not reveal features of thrombotic microangiopathy (which was the initial working diagnosis given the hemolytic anemia and thrombocytopenia), but rather showed features of MCD. The patient had a known history of HCV infection with a detectable HCV RNA at the time of presentation, and he had not received prior interferon-based therapy. The hematologic abnormalities and acute kidney injury resolved spontaneously. Oral prednisone was initiated for persistent proteinuria and edema, with resolution of the proteinuria 1 month later. No further follow up was provided, and there was no information on subsequent treatment of the HCV infection [13]. In 2012, Stokes et al. [6] reported the case of a 58-year-old African-American man with a history of urethral stricture and rheumatoid arthritis (on chronic prednisone therapy) who presented with abdominal pain, weight gain, and lower extremity edema. He had nephrotic range proteinuria and acute kidney injury. His kidney biopsy revealed features of MCD. He was also found to have a reactive HCV antibody titer and a high HCV RNA load. He was discharged on oral prednisone therapy (1 mg/kg/day), and at 3 months, his serum creatinine had normalized, the nephrotic syndrome was in remission, and as such, the prednisone taper was initiated. Although he was scheduled to be evaluated in the hepatology clinic for interferon-based therapy, no further information was provided regarding HCV therapy or further tapering of prednisone [6]. In 2018, Chowdhury et al. [14] reported the case of a 44-year-old (ethnicity not reported) man with known HCV infection, who presented with lower extremity edema and was found to have nephrotic range proteinuria (12.1 g/day). There was no mention of prior exposure to interferon therapy or non-steroidal anti-inflammatory drugs (given his history of rheumatoid arthritis). The kidney biopsy revealed diffuse podocyte effacement on electron microscopy, in support of a diagnosis of MCD. However, the biopsy also exhibited mild increase in mesangial matrix and minimal segmental increase in cellularity on light microscopy, and there was mild mesangial staining for IgA, with trace amounts of C3, κ and λ light chains on immunofluorescence microscopy, raising the possibility of a concomitant mild IgA nephropathy. The patient was initiated on oral prednisone (1 mg/kg/day). Unfortunately, no information was provided on his kidney function and HCV RNA titer at the time of his initial presentation, and there was no follow-up on the status of the nephrotic syndrome and potential HCV treatment [14]. Similarly, our patient presented mostly with signs of volume overload, and her initial proteinuria was so severe that it could not be quantified using the spot urine total protein-to-creatinine ratio. Her acute kidney injury was due to acute tubular necrosis and was severe enough to require short-term dialysis. 

Compared to children, adults with MCD tend to present more frequently with hypertension, hematuria, and acute kidney injury [15]. Renal impairment is noted in 20-30% of adults with MCD, with up to 25% requiring dialysis [16]. Older age and the degree of proteinuria and hypoalbuminemia are risk factors for renal impairment in adults with MCD [3, 16]. Similarly to our patient, acute tubular necrosis is frequently noted in adults with MCD and acute kidney injury [15, 16]. In adults, acute tubular necrosis has been recently hypothesized to be secondary to endothelin-1 mediated vasoconstriction at the onset of proteinuria with a subsequent decrease in renal perfusion, further perpetuated by diuretic-induced hypovolemia, arteriolar nephrosclerosis, and exposure to nephrotoxic agents [16, 17]. 

HCV-related glomerulopathies are mostly due to cryoglobulins and non-cryoglobulin-immune complex deposition. A direct viral cytopathic effect has also been proposed as a contributor to the pathogenesis, particularly in the case of membranous nephropathy and focal segmental glomerulosclerosis [5, 18, 19]. While in the case of our patient, a direct podocyte injury from HCV cannot be excluded, we postulate that HCV triggered the MCD indirectly through immune dysregulation or stimulation, as seen in patients who develop MCD in association of vaccination and other viral infections. Indeed, the electron microscopy did not reveal any viral inclusions to suggest a direct viral podocytopathy. Moreover, the immunofluorescence microscopy revealed “fine-dusting” of IgG deposits on the cell surface of the podocytes, a finding reported in a subset of patients with MCD that might indicate an auto-immune process against nephrin. We can only postulate as to whether HCV triggered the production of anti-nephrin antibodies [20]. This explains the initial drastic improvement in kidney function and proteinuria with prednisone before initiating therapy with DAAs. 

Nonetheless, with the advancement of DAAs, the postulation that the virus itself might be a triggering factor rather than a mere coincidence has important potential implications for treating HCV-associated MCD. Indeed, antiviral therapy is the cornerstone in the treatment of HCV-associated glomerular diseases. Among patients with HCV-associated mixed cryoglobulinemia, many who achieve sustained virologic remission with DAAs also demonstrate complete or partial remission of the glomerular disease [21]. Although our patient remained inadvertently on low-dose prednisone longer than the planned 16-week taper, she successfully completed a 12-week course of ledipasvir/sofosbuvir. We can only speculate as to whether the sustained virologic remission achieved by the DAAs resulted in a sustained remission of her nephrotic syndrome, as seen in other histopathologic types of HCV-associated glomerular diseases. Although our patient was not very compliant with her clinic visits, she had no evidence of albuminuria 2 years later, despite discontinuation of steroids, in support of a sustained remission of her nephrotic syndrome. 

## Conclusion 

In conclusion, HCV-associated MCD is a very rare presentation in treatment-naïve patients. An optimal treatment strategy is not established given the rarity of the disease. However, as suggested in our case report, sustained remission of the nephrotic syndrome can be achieved with a combined therapeutic approach, namely a 16-week course of prednisone for the proteinuria coupled with a 12-week course of DAAs for the HCV viremia. Whether sustained virologic remission achievement should influence the optimal duration of prednisone therapy needs to be elucidated. 

## Authors’ contributions 

JA, HA, DD drafted the manuscript. JA, CN, and BJ were the physicians directly involved in the care of the patient. HR was the nephropathologist who reviewed the kidney biopsy and generated the figures. CN, HR, and BJ revised the manuscript and performed the literature review. JA and BJ take responsibility that this case has been reported honestly, accurately, and transparently, and accept accountability for the overall work by ensuring that questions about the accuracy or integrity of any portion of the work are appropriately investigated and resolved. 

## Funding 

The authors report no external funding source for this report. 

## Conflict of interest 

The authors report no conflict of interest. 

**Table 1.** Summary of the clinical, laboratory, and treatment features of HCV-associated minimal change disease in anti-viral treatment-naïve patients.

Author	Year	Age	Sex	Ethnicity	Clinical presentation	Initial serum creatinine (mg/dL)	Urine total protein excretion	HCV RNA level (10^6^ IU/mL)	HCV genotype	MCD treatment	HCV Treatment	Kidney-related parameters at last follow-up	Sustained virologic response
Aoyama et al. [13]	2007	49	Male	Japanese	Acute onset anasarca	2.33	13.6 gm/day	3.1	NR	Prednisone (40 mg/day); duration not provided	NR	Proteinuria 0.18 g/day and serum creatinine 0.92 mg/dL, at 1 month	NR
Stokes et al. [6]	2012	58	Male	African American	Abdominal pain, dark-colored urine, lower extremity edema, and 30-pound weight gain	1.70	18 gm/day	>27	NR	Prednisone (1 mg/kg/day); taper initiated after 3 months; duration of taper not provided	Evaluation regarding candidacy for interferon-α therapy (no further details provided)	Proteinuria 0.45 g/g and serum creatinine 0.89 mg/dL, at 3 months	NR
Chowdhury et al. [14]	2018	44	Male	NR	Lower extremity edema	NR	12.1 gm/day	NR	NR	Prednisone (1 mg/kg/day); treatment duration not provided	NR	NR	NR
Alhaddad et al., present case	2024	67	Female	White	Bilateral knee pain (following a fall), and anasarca	7.0	> 600 mg/dL	1.78	1b	Pulse methylprednisolone (4 days) followed by prednisone 60 mg daily, tapered in the outpatient setting	Ledipasvir/sofosbuvir	Urine albumin to creatinine ratio < 30 mg/gm, and serum creatinine 0.79 mg/dL, at 24 months	Achieved

HCV = hepatitis C virus; NR = not reported.
